# Preparation of Chitosan-Based Emodin Antimicrobial Functional Films and Application in the Preservation of Chilled Pork

**DOI:** 10.3390/foods15030490

**Published:** 2026-02-01

**Authors:** Xu Qiu, Dongxu Liu, Guoyuan Xiong, Junying Wang, Shengming Zhao, Baoshi Wang, Yanyan Zhao, Ligong Zhai

**Affiliations:** 1 College of Food Science and Engineering, Anhui Science and Technology University, Chuzhou 239001, China; yjs2023238@ahstu.edu.cn (X.Q.); liudongxu20011211@163.com (D.L.); guoyuanx979@163.com (G.X.); wangjy@ahstu.edu.cn (J.W.); zhaoshengming2008@126.com (S.Z.); wangbaoshifsd@126.com (B.W.); zhaoyanyan@ahstu.edu.cn (Y.Z.); 2 Key Laboratory of Functional Agriculture and Functional Foods, Anhui Science and Technology University, Chuzhou 239001, China

**Keywords:** photodynamic inactivation, emodin, chitosan, antimicrobial activity, food packaging, chilled pork

## Abstract

This study aimed to develop natural, safe, and effective antimicrobial packaging materials for extending the shelf life of chilled pork during refrigeration. Emodin-chitosan (Em-Cs) composite films with varied concentrations were developed by combining the casting method with photodynamic inactivation technology, utilizing chitosan as the matrix and emodin as the functional photosensitizer for active packaging. The optical, mechanical, and barrier properties of the composite films were examined. The inhibitory effects of the samples on *Escherichia coli*, *Salmonella* Derby, *Staphylococcus aureus*, and *Pseudomonas fragi* under 450 nm blue light irradiation were evaluated. The results demonstrated that the Em-Cs composite film exhibited excellent transparency, mechanical strength, and water barrier properties, with good compatibility between emodin and chitosan. Under light irradiation, the composite film generates reactive oxygen species (ROS), whose bactericidal efficacy depends on the concentration of emodin and the duration of light exposure. When applied to chilled pork packaging, this composite film inhibited bacterial growth within the meat for 10 days, effectively retarding pH increase, lipid oxidation, and volatile basic nitrogen accumulation. The present study proposes a novel methodology for the application of photodynamic technology in the context of food preservation, and it presents a new type of natural antimicrobial packaging material for the preservation of chilled pork.

## 1. Introduction

The term “chilled pork” is used to describe pork products that undergo a series of processes, including the removal of acid, cutting, storage, transportation, and sale at a controlled low temperature of 0–4 °C [1]. The purpose of these processes is to maximize the preservation of the meat’s flavor, quality, and nutritional value [1]. However, chilled pork is highly susceptible to microbial contamination due to increased enzyme activity, fat content, and water activity, resulting in a shorter shelf life [2]. The addition of preservatives to food packaging is an effective means to extend the shelf life of foods. Concerns regarding the toxicological risks associated with traditional chemical preservatives have prompted the food industry to explore natural alternatives [3]. Consequently, the development of natural antimicrobial agents and antimicrobial packaging films has emerged as a key research area for prolonging the shelf life of chilled pork products [4]. The core principle of preserving chilled pork lies in suppressing microbial proliferation and enzymatic activity through low temperatures, while minimizing moisture loss and oxidation. Fresh pork can maintain its freshness for 5–7 days when stored at 4 °C without vacuum packaging [5]. When modified atmosphere packaging with high CO_2_ concentration is employed alongside an uninterrupted 4 °C cold chain, the shelf life of chilled pork can be further extended to 7–10 days [6].

Photodynamic inactivation (PDI) is a non-thermal microbial inactivation technique that uses light, photosensitizers, and oxygen to inhibit foodborne pathogens [7]. PDI comprises three key components: a photosensitizer (e.g., curcumin), a light source (e.g., light-emitting diodes), and oxygen [8]. Upon excitation of the photosensitizer by specific light, reactive oxygen species (ROS) are generated to attack cellular DNA, RNA, proteins, and other components, ultimately inducing cell death [9]. Reports indicate its application in preserving aquatic products such as oysters, shrimp, and clams, extending their storage life [10]. It was reported that the antioxidant properties and flexibility of Chitosan (Cs)-based film were increased as the curcumin content increased, in which the DPPH free radical scavenging rate and elongation at break were up to 56.82% and 49.45%, respectively [11]. The Cs film incorporating aloe emodin (AE) exhibited outstanding antibacterial activity, with significantly enhanced elongation at break, water vapor permeability, and oxygen permeability compared to pure chitosan films [12]. However, photosensitizers such as curcumin possess strong coloring properties that adversely affect food color quality, thereby limiting the widespread adoption of this technique [13]. Incorporating natural photosensitizers into films and mediating photodynamic technology represents a promising new approach for food preservation [8].

Emodin (1,3,8-trihydroxy-6-methylanthraquinone) is a natural anthraquinone compound primarily extracted from rhubarb, *Polygonum cuspidatum*, and *Polygonum multiflorum* [14]. It exhibits diverse pharmacological activities, including anti-inflammatory, anti-diabetic, anti-cancer, and antibacterial effects [15]. Previous research has demonstrated its broad-spectrum activity against Gram-positive bacteria [14,16]. However, as a novel natural photosensitizer, emodin’s photodynamic effects remain poorly documented, with only one investigation having examined its photodynamic antibacterial activity against *Escherichia coli* and *Staphylococcus aureus* [17].

As a non-thermal microbial inactivation technique, photosensitizer-mediated PDI is regarded as a highly promising microbial inactivation strategy in the food sector [18]. Only one study has investigated the PDI of Em [16], alongside two studies on aloe emodin (AE) in food preservation [12,19]. To date, no studies have attempted to prepare chitosan-based composite films incorporated with emodin (Em) and synergize them with photodynamic technology for chilled pork preservation. Notably, chitosan is widely recognized as an ideal film-forming matrix for photosensitizers due to its excellent biocompatibility and film-forming ability [11].

In this study, we innovatively integrate emodin (Em) with chitosan (Cs) to fabricate Em-Cs composite films via the cast film method, constructing a film-photodynamic microbial inactivation system. The Em-Cs film does not contact chilled pork during preservation. This work fills the research gap of using emodin-chitosan composite films in PDI-mediated food preservation, resolves the coloration issues associated with previous photosensitizing agents, and provides a novel natural antimicrobial packaging solution for chilled pork—with the potential to overcome the limitations of existing photodynamic preservation technologies and conventional chitosan-based films.

## 2. Materials and Methods

### 2.1. Materials

Chitosan (Cs, food grade, deacetylation degree ≥ 95%, viscosity 100–200 mPa·s) was obtained from Shanghai McLean Biochemical Technology Co., Ltd. (Shanghai, China). Emodin (Em, CAS: 518-82-1, purity ≥ 90.0%) was offered by Sangon Biotech Co., Ltd. (Shanghai, China). 2′,7′-Dichlorofluorescin diacetate (DCFH-DA) was procured from Shanghai Beyotime Biotechnology Co., Ltd. (Shanghai, China). Disposable plastic Petri dishes (90 mm × 90 mm × 15 mm) were acquired from Changde Bickman Biotechnology Co., Ltd. (Changde, China). Chilled pork was brought from a local supermarket (Tenderloin (*Psoas major*), from pigs slaughtered the previous day, Chuzhou, China). Experimental strains: *Escherichia coli* ATCC 25922, *Salmonella* Derby CMCC 50719, *Staphylococcus aureus* CICC 10788, and *Pseudomonas fragi* CGMCC 1. 3349 were all stored in our laboratory.

### 2.2. Preparation of Photosensitive Composite Film

Cs and Em-Cs composite films were prepared via the cast-film method, which was slightly modified from the approach described by Ashrafi et al. (2018) to formulate the film-forming solution (FFS) [20]. Based on the Em concentration, the films were designated as Pure Cs, Em^1^-Cs, Em^2^-Cs, and Em^3^-Cs, respectively [21].

**Figure 1:**
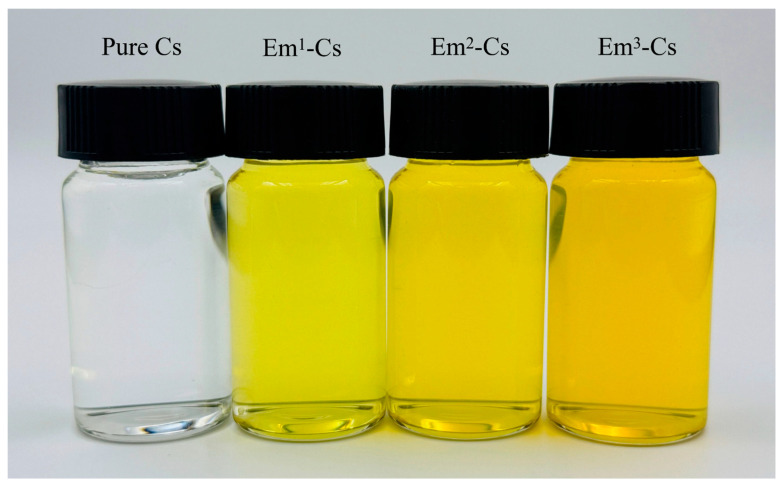
Film-forming solutions (FFS) with different concentrations of Emodin.

The chitosan powder was dissolved in an acetic acid solution (1% *v*/*v*), and it was then stirred continuously at 90 °C for 10 min using a magnetic stirrer to prepare the chitosan film-forming solution (1% *w*/*v*). Glycerol (0.8% *v*/*v*, plasticizer) and a defined volume of emodin solution were added to the chitosan film-forming solution to achieve emodin concentrations of 0, 100, 150, and 200 μmol/L within the film-forming solution system, as illustrated in Figure 1. The mixture was stirred for 30 min in the dark to achieve homogeneity. Following mixing, the film-forming solution was subjected to ultrasonic degassing for 15 min using an ultrasonic device (UA10MFDN, WIGGENS, Straubenhardt, Germany) to eliminate impurities and bubbles. 15 mL of Cs/Em-Cs FFSs were dispensed into disposable plastic petri dishes (90 mm × 90 mm × 15 mm) and dried at 40 °C for 8 h. After cooling naturally to room temperature, the films were peeled off and conditioned for 48 h at 24 ± 1 °C and 50% RH (relative humidity), yielding Pure Cs and Em-Cs composite films. The films were subsequently stored in a dark, dry environment for all subsequent characterization experiments.

### 2.3. Determination of the Properties of Cs Film and Em-Cs Films

#### 2.3.1. Optical Properties

The color of the film was measured using an NR110 color difference meter (Measurement aperture size of φ4 mm, optical geometry of 8°/d (8° illumination/diffuse viewing), CIE 10° standard observer angle, and D65 standard light source (LED blue light excitation), Threenh Technology Co., Ltd., Shenzhen, China). Results were expressed as Hunter L* (lightness), a* (red/green), and b* (yellow/blue). A white standard plate (L_0_ = 97.34, a_0_ = −0.15, b_0_ = 0.37) was used, with five measurements taken per sample. The total color difference (ΔE) was calculated as follows (1):

(1) $$\Delta E=\sqrt{{\left(L*-L_{0}\right)}^{2}+{\left(a*-a_{0}\right)}^{2}+{\left(b*-b_{0}\right)}^{2}}$$

#### 2.3.2. Opacity Value

The UV-visible spectra of the film samples were recorded using a UV spectrophotometer (UV-1800, Shimadzu, Tokyo, Japan) [22]. The opacity value of the film was calculated as follows (2):

(2) $$Opacity\;value=\frac{-\log T_{600}}{x}$$

where *T*_600_ represents the absorbance of the film at 600 nm; *x* is the film thickness (mm).

#### 2.3.3. Thickness and Mechanical Properties

The thickness of the film samples was obtained by averaging five random points on the film using a digital helical micrometer (Mitutoyo Manufacturing Corporation, Kawasaki, Japan).

Tensile strength (TS) and Elongation at break (EB) of the film samples were determined using an automatic tensile tester (XLW-EC, PARAM, Jinan, China). Five identical film samples were cut into strips (100 mm × 15 mm) with an initial marking distance of 50 mm and a tensile speed of 50 mm/min [23].

#### 2.3.4. Water Contact Angle (WCA), Moisture Content (MC), Water Solubility (WS), and Water Vapor Permeability (WVP)

The water contact angle (WCA) of the film was measured using a video contact angle measuring instrument (JY-82C, Chengde Dingsheng Testing Machine and Inspection Equipment Co., Ltd., Chengde, China) to evaluate its wettability. A 2 μL water droplet was placed on the sample surface, and the WCA was subsequently recorded.

Film samples (2 × 2 cm, M_1_) were dried in an oven at 105 °C to reach a constant weight (M_2_). They were then immersed in a sealed conical flask containing 50 mL of distilled water and stored at ~25 °C for one day. After filtering out the excess water, the samples were dried at 105 °C to constant weight (M_3_). The MC and WS (%) of the membrane samples were calculated as follows (3) [24]:

(3) $$MC\left(\%\right)=\frac{M_{1}-M_{2}}{M_{1}}\times 100\;WS\left(\%\right)=\frac{M_{2}-M_{3}}{M_{2}}\times 100$$

Water vapor permeability (WVP) was determined by the gravimetric method with some modifications with reference to Wang et al., 2022 [25]. The film samples were covered with the circular opening of a glass weighing flask (40 mm × 70 mm) containing 20 mL of deionized water and sealed tightly with a sealing film to prevent water vapor leakage. The glass weighing flasks were placed in a brown desiccator containing silica gel and kept at a room temperature of 25 °C. The glass weighing flasks were monitored at 2-h intervals. The weight change of the glass weighing flasks was monitored every 2 h. Water vapor transmission rate (WVTR) and WVP are obtained using the following Formula (4) [26]:

(4) $$WVTP=\frac{∆w}{A\times ∆t}\;WVP=\frac{WVTR\times L}{∆P\times ∆RH}$$

where Δ*W*/Δ*t* denotes the change in mass over time (g/h); *A* is the area of the test film (m^2^); *L* is the thickness of the average film (m); Δ*P* denotes the difference in water vapor pressure between the two sides of the film (kPa); and Δ*RH* is the difference in relative humidity between the two sides of the film (%).

#### 2.3.5. Scanning Electron Microscopy (SEM)

The surface and cross-sectional morphology of the membrane samples were obtained using a scanning electron microscope (Sigma 300, ZEISS, Oberkochen, Germany). The films were coated with a 5 nm gold color, and the images were observed in low vacuum mode at an operating voltage of 20 kV and 10 kV, respectively [27].

#### 2.3.6. Fourier Transform Infrared Spectrometry (FTIR)

The spectra of Em and the film samples were acquired using the KBr module of a Fourier Transform Infrared Spectrometer (Nicolet iS20, Thermo Fisher, Waltham, MA, USA). FTIR spectra were recorded at a resolution of 4 cm^−1^ and scanned 32 times within the wavenumber range of 4000–400 cm^−1^.

#### 2.3.7. The X-Ray Diffractions

Wide-angle XRD patterns of all films were recorded using an X-ray diffractometer (SmartLab SE, Rigaku, Tokyo, Japan) [24]. The working conditions were set in the range of 5 to 50° diffraction angle (2θ), and the scanning speed was 2°/min.

#### 2.3.8. Thermal Performance

The thermal properties of membrane samples were determined using a thermal analyzer (TGA/DSC3+, Mettler Toledo, Greifensee, Switzerland). Membrane samples (4–5 mg) were sealed in standard aluminum pans and heated at a rate of 10 °C/min within the range of 30–800 °C, with nitrogen employed as the protective atmosphere.

### 2.4. Light Sources and Cultured Bacteria

The light source used in the following experiment was blue LEDs (455 ± 5 nm, 15 cm, 40 W, Xuzhou Aijia Electronic Technology Co., Ltd., Xuzhou, China). Standard strains stored in glycerol tubes at −80 °C were inoculated into Luria-Bertani broth (LB) medium (50 mL). Cultures were shaken at 37 °C for 12 h. while *P. fragi* was inoculated into Nutrient Agar (NB) medium. Cultures were shaken at 30 °C for 24 h. The bacterial culture reaching a density of approximately 10^8^ CFU/mL was diluted with sterile physiological saline to ~10^7–8^ CFU/mL and used immediately for the experiment.

### 2.5. Photodynamic Effect of Em-Cs Film Combined with 450 Nm Light on Bacteria

#### 2.5.1. Investigation of Different Concentrations of Em-Cs Film and Irradiation Duration

First, the prepared composite membranes were cut into 2 × 2 cm squares and placed in a six-well plate. Subsequently, 100 μL of a tenfold diluted bacterial suspension (10^8^–10^9^ CFU/mL, diluted with 0.85% saline) was added to the surface of each membrane. After exposure to the LED illumination system for varying durations (20 min, 40 min, 60 min), 900 μL of 0.85% saline solution was added to the membrane surface, and the membranes were washed several times [28]. The mixture was aspirated, and the membrane was transferred into a 1.5 mL centrifuge tube using forceps. The centrifuge tube was then incubated on an incubator shaker for 5 min. After serial dilutions, appropriate concentration gradients were selected. Subsequently, 100 μL of the diluted suspension was pipetted and spread onto plate count agar (PCA). The agar plates were incubated for 24 h (*E. coli*, *S.* Derby, *S. aureus*) or 36–48 h (*P. fragi*) to evaluate the antimicrobial activity of Em-mediated 450 nm LED light against the four bacterial strains. Live bacterial counts were recorded using the plate count method, with results expressed as log_10_ CFU/mL.

For comparative purposes, Cs membranes with and without light exposure (L − E−) served as negative controls, light-exposed Cs membranes (L + E−) as light exposure controls, and non-light-treated Em-Cs composite membranes (L − E+) as Em controls. The experimental group comprised light-exposed Em-Cs composite membranes (L + E+) [29].

#### 2.5.2. Production of ROS Inside Bacterial Cells

Using 2′,7′-Dichlorofluorescin diacetate (DCFH-DA) as a probe, ROS production within bacterial cells was detected via inverted fluorescence microscopy (AIRP-IR500FL, Kunshan Alp Electronics Co., Ltd., Kunshan, China). Membranes were cut into 2 × 2 cm pieces and placed in six-well plates. Subsequently, 100 μL of bacterial suspension was added to the Em-Cs composite membrane and incubated in darkness for 30 min, followed by treatment with 450 nm blue light irradiation for 20 min. Thereafter, 900 μL of 0.85% physiological saline was added to the membrane surface, and the surface was washed several times. Then, the resulting mixture was aspirated into a 1.5 mL centrifuge tube. The tube was centrifuged to collect the bacterial pellet, which was then washed twice with sterile phosphate-buffered saline (PBS). Next, the bacterial pellet was resuspended in sterile PBS, and 200 μL of 20 μM DCFH-DA solution was added to the suspension. The bacterial suspension was incubated in the dark for 30 min, after which the incubated bacteria were washed again with sterile PBS. Subsequently, the washed bacteria were resuspended in 200 μL of physiological saline. Finally, 10 μL of the prepared bacterial suspension was pipetted onto a microscope slide, the suspension was covered with a coverslip, and the bacterial fluorescence was observed under an inverted fluorescence microscope.

### 2.6. Effect of Em-Cs Film Combined with 450 Nm Light Source Treatment on the Preservation of Chilled Pork

#### 2.6.1. Chilled Pork Preservation Experiment

Fresh chilled pork was cut into uniform pieces weighing approximately 15 g each. The chilled pork samples were placed in disposable Petri dishes. Samples in the control group were left uncovered, while those in the remaining three experimental groups were covered with commercially available PE cling film (Low-Density Polyethylene, purchased from a local supermarket, Thickness: 0.008–0.015 mm), Cs film, and Em-Cs composite film, respectively. During the covering process, direct contact between the film and the chilled pork samples was minimized. After covering the pork samples with the Em-Cs composite film, they were irradiated with a 450 nm LED light source (lighting power 40 W, film-to-light distance 15 cm, irradiation time 60 min). Following treatment, all samples were stored at 4 °C. Samples were retrieved at 0, 2, 4, 6, 8, and 10 days for quality parameter analysis, investigating the effects of different packaging treatments on pork quality indicators during storage.

#### 2.6.2. pH Measurement of Chilled Pork

The pH determination of the samples was performed according to the method described by Zeng et al. (2023) [30]. 5 g of chilled fresh meat was weighed and thoroughly mixed with deionized water (1:9, m:m). The prepared mixture was homogenized until it became uniform in texture. The homogenized mixture was allowed to stand undisturbed for 30 min. The supernatant of the mixture was collected, and its pH value was measured using a pH meter (FE28, LE438, Mettler Toledo, Greifensee, Switzerland).

#### 2.6.3. Thiobarbituric Acid Reactive Substances (TBARS) Measurement of Chilled Pork

The determination of TBARS values was performed according to the method described by Sun et al. (2021) [31]. A 5 g portion of the minced meat sample was weighed. 50 mL of trichloroacetic acid mixture (containing 0.1% EDTA) was added to the weighed sample. The mixture was homogenized at 8000 r/min for 30 s. The homogenized mixture was filtered, and the resulting filtrate was allowed to cool to room temperature. Subsequently, 5 mL of the cooled filtrate was pipetted and thoroughly mixed with 5 mL of 2-thiopropionic acid solution (20 mmol/L). The mixed solution was incubated in a 90 °C water bath for 30 min, then cooled back to room temperature. The absorbance of the incubated solution was measured at a wavelength of 532 nm, and the TBARS value was calculated and expressed as milligrams of malondialdehyde (MDA) per kg of meat.

#### 2.6.4. Total Viable Counts (TVC) Measurement of Chilled Pork

The TVC measurements were performed for microbiological analysis according to the method of Feng et al. (2016) [32]. On each sampling day, chilled fresh meat samples were thoroughly minced. 5 g of the minced meat sample was weighed and transferred into a sterile homogenization bag pre-filled with 45 mL of sterile physiological saline. The mixture inside the bag was homogenized using a paddle-type homogenizer for 1–2 min, producing a 1:10 sample homogenate. From each sample, 1 mL was taken for serial dilution. Three appropriate dilution levels (100 μL each) were spread onto PCA plates in triplicate. After incubation at 37 °C for 24–48 h, the colonies were counted using the plate count method. Results were expressed as log_10_ CFU/g.

#### 2.6.5. Total Volatile Basic Nitrogen (TVB-N) Measurement of Chilled Pork

The TVB-N value of pork was determined in accordance with the Chinese National Standard GB 5009.228-2016 [33]. 10 g of chilled fresh pork sample was homogenized in 50 mL of distilled water, and the mixture was soaked for 30 min prior to filtration. Bromothymol blue indicator was added to the central chamber of the diffusion cell. Simultaneously, 1 mL of the filtrate and 1 mL of saturated sodium carbonate solution were added to the outer chamber of the diffusion cell. The contents in the outer chamber were thoroughly mixed, and the diffusion cell was then placed in a sealed environment. The sealed diffusion cell was incubated at 37 ± 1 °C for 2 h. Subsequently, the absorption solution was titrated with a standard hydrochloric acid solution until the titration endpoint was reached. Triplicate measurements were performed for each sample to determine the TVB-N value. The volatile basic nitrogen content, expressed as milligrams of nitrogen per 100 g of sample, was calculated using the following Formula (5):

(5) $$TVB-N=\frac{C\times \left(V-V_{0}\right)\times 14\times 5000}{W}$$

TVB-N denotes the total volatile basic nitrogen content in the sample, expressed in milligrams per 100 g; *V* represents the volume of hydrochloric acid consumed; *V*_0_ denotes the volume of hydrochloric acid consumed during the blank titration, measured in milliliters; *W* indicates the weight of the meat sample, expressed in grams; *C* signifies the concentration of hydrochloric acid, measured in grams per mole.

### 2.7. Data Analysis

All the experiments described were performed at least three times, and the results were expressed as mean ± standard deviation. The experimental data were analyzed using SPSS (SPSS 27. 0 Software, Inc., Chicago, IL, USA) software. One-way Analysis of Variance (ANOVA) was used to compare the differences between the means of the two groups (*p* ≤ 0.05). Statistical data were plotted using Origin 2024 drawing software.

## 3. Results and Discussion

### 3.1. Optical Properties of the Em-Cs Composite Films

The optical properties of food packaging films indirectly influence the appearance of packaged foodstuffs, thereby affecting consumer perception [34]. Images, color values (L*, a*, b*, and ΔE), and opacity of film samples are listed in Table 1. Pure Cs films are transparent and colorless, gradually yellowing with increasing Em concentration. The L* value of the films remained unaffected by Em, varying between 94.43 and 92.54, close to the control white sheet, indicating all films were transparent. Film opacity increased with rising Em concentration. However, opacity at 600 nm never exceeded 5, demonstrating good transparency consistent with the L* values. The a* values for pure Cs and Em-Cs composite films were negative, indicating the absence of red in Em-Cs composite films. The b* value indicates the blue-to-yellow hue of the film, while Δ*E* demonstrates the color difference between the white board and the Em-Cs composite film. As Em appears yellow after absorbing blue light, the b* value increases with the addition of Em. Consequently, the total color difference (Δ*E*) of the Em-Cs composite film increases with rising Em concentration, exhibiting a pronounced yellow hue. The L* value of Em-Cs composite films varies with increasing Em content (94.43 ± 0.48~92.54 ± 1.03) significantly lower than that reported by Wen et al. (2023) for chitosan-curcumin films (99.99 ± 0.01~81.005 ± 0.706) [35], as well as Wang et al. (2022) as reported Gelatin/Chitosan-Curcumin Film (93.38 ± 0.07~93.38 ± 0.07) [25]. Opacity is also lower than that of these two films.

### 3.2. Thickness and Mechanical Properties of the Em-Cs Composite Films

The thickness and mechanical properties of the film samples are presented in Table 2. Film thickness may influence mechanical properties [36]. Incorporation of Em into Cs films increased the thickness from 0.023 mm to 0.028 mm. This network, when cast onto plastic substrates, yields films whose thickness is directly influenced by the Em content in the formulation matrix. This correlation, which confirms that adding solid components enhances film thickness, is corroborated by the work of Haghighi et al. (2020) [37].

In terms of mechanical properties, sufficient mechanical strength and ductility are crucial for food packaging films [38], both tensile strength and elongation at break were evaluated, with results presented in Table 2. Following Em addition, all two-parameter values increased across all films. The TS values of the Em-Cs composite films approached those of common plastic films widely used in food packaging, such as high-density polyethylene (22–31 MPa), but remained below those of polypropylene (31–38 MPa) and polystyrene (45–83 MPa) by Farhan & Hani (2017) [39]. The TS of Em^3^-Cs film reached 26.32 ± 1.25 MPa, which is higher than that of chitosan-aloe emodin films (25.43 ± 1.57 MPa) reported by Yang et al. (2024) and chitosan-curcumin films (18.12 ± 0.31 MPa) by Wang et al. (2022) [12,25]. The interaction between Cs and Em likely contributed to the enhanced mechanical properties of the films, with multiple factors potentially influencing this outcome, such as the degree of deacetylation and molecular weight of Cs, or the drying conditions of the film-forming solution, amongst others.

### 3.3. MC, WS, WVP, and WCA of the Em-Cs Composite Films

The water sensitivity of biodegradable films has a significant impact on their application [40]. Consequently, measuring the film’s water sensitivity is necessary to verify its suitability for food packaging [22]. Table 2 presents the film’s moisture content (MC), water solubility (WS), water contact angle (WCA), and water vapor permeability (WVP).

Following the Em addition, the moisture content of the film decreased. The water solubility of the film, defined as the soluble dry matter content after 24 h of immersion in water, also decreased with increasing Em addition. The pure Cs film exhibited the lowest WS value, whilst the Em^1^-Cs composite film demonstrated the highest WS value (*p* < 0.05). This is attributed to the lower affinity of high-concentration Em towards water molecules.

**Figure 2:**
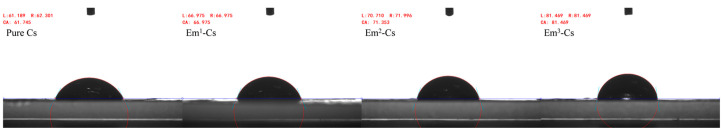
Water contact angle of Pure Cs film and Em^1^-Cs, Em^2^-Cs, and Em^3^-Cs composite films. Pure Cs: Chitosan film without emodin; Em^1^-Cs film: Chitosan composite film with 100 μmol/L emodin solution; Em^2^-Cs film: Chitosan composite film with 150 μmol/L emodin solution; Em^3^-Cs film: Chitosan composite film with 200 μmol/L emodin solution.

The WCA reflects the affinity of the composite film for water [41]. As shown in Figure 2, the results showed all films exhibited a tendency towards hydrophilicity, yet the WCA value increased with the addition of Em, indicating reduced hydrophilicity. Consequently, it is anticipated that the film samples will not react with foods possessing high moisture content. The film’s water barrier properties are also crucial for determining the extended shelf life of packaged foods [42]. When evaluating a film’s water barrier properties, water vapor permeability (WVP) must also be considered. For effective shelf-life extension, the film must provide an adequate barrier against environmental water vapor, making a low WVP imperative [43]. As the Em concentration increases, the WVP value of the film samples decreases from 3.63 × 10^−13^ to 1.92 × 10^−13^ g/(m·s·Pa), thereby enhancing water barrier performance. The WVP of Em^3^-Cs films was 1.83 × 10^−13^ g/(m·s·Pa), significantly lower than that of chitosan-cellulose composite films (1.92 × 10^−13^ g/(m·s·Pa)) by Su et al. (2021), indicating better water barrier capacity [44]. The observed effect was ascribed to the combined effect of the increased film thickness from the Em incorporation and the cross-linking interaction between Cs and Em, leading to a reinforced film matrix.

### 3.4. Characterization of the Em-Cs Composite Films

#### 3.4.1. SEM

**Figure 3:**
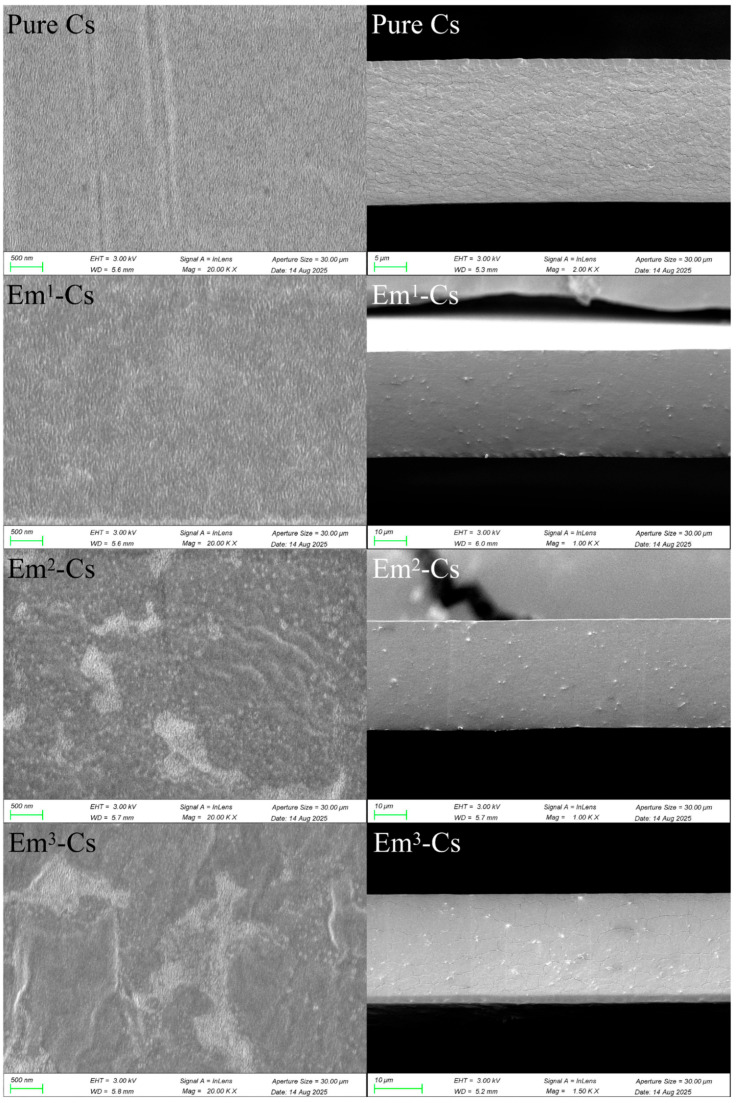
Scanning electron microscope (SEM) images of the surface and cross-section of the Chitosan and Emodin-Chitosan composite films.

The microstructure of the film may influence its mechanical properties due to interactions between the film-forming substrates [44,45]. Surface and cross-sectional views of the pure Cs film and Em-Cs composite film are shown in Figure 3. The film surface appears smooth, uniform, and void-free; however, with increasing Em concentration, the film exhibits slight irregularities and minor agglomeration under microscopic examination. Nevertheless, no discernible morphological differences exist between pure Cs and Em-Cs composite films, indicating negligible surface modification by Em addition and uniform dispersion of Em throughout the Em-Cs film-forming solution. This result is in full agreement with the earlier report by Su et al. (2021), which found no significant variation among chitosan film samples following riboflavin incorporation [44]. These investigations collectively demonstrate that emodin can also be uniformly distributed within the film-forming matrix, with Cs and Em exhibiting good compatibility for hybrid membrane formation.

#### 3.4.2. FTIR

**Figure 4:**
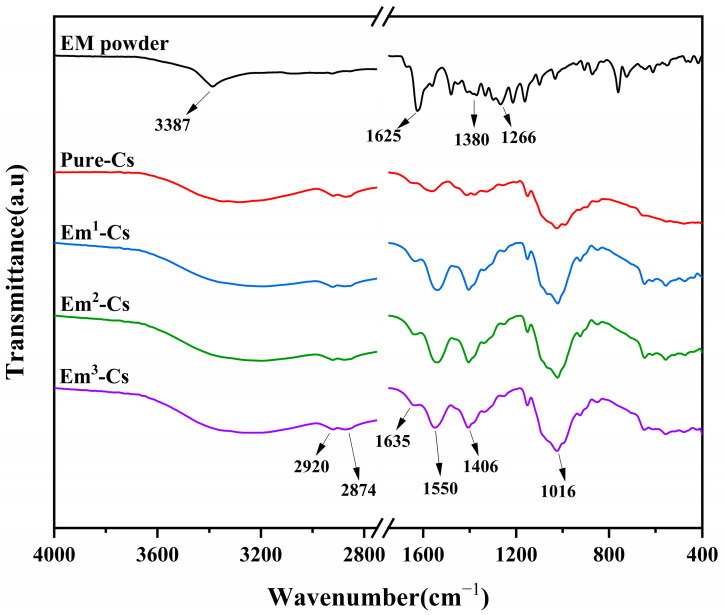
Fourier transform infrared (FTIR) spectra of Chitosan and Emodin-Chitosan composite films.

FTIR facilitates understanding of the structural properties of the membrane by characterizing the intermolecular interactions between Cs and Em [46]. The spectra of Em powder, Cs membrane, and Em-Cs composite membrane are shown in Figure 4. The broad band between 3500 and 3000 cm^−1^ is attributed to the stretching vibrations of the -OH group [47]. This is characteristic of hydroxyl groups involved in intramolecular and intermolecular hydrogen bonding. The characteristic absorption bands of Em powder at 1625, 1380, and 1266 cm^−1^ correspond to the stretching vibrations of the carbonyl group, the -CH_3_ group, and C-O bonds (with O-H bending), respectively [47].

The adjacent double peaks in the spectra of Cs membranes and Em-Cs composite membranes at ~2920 and 2874 cm^−1^ are attributed to the stretching vibrations of -CH_2_ and -OH groups [47]. The peak at 1635 cm^−1^ relates to hydroxyl stretching vibrations, whilst those at 1550, 1406, and 1016 cm^−1^ correspond to asymmetric stretching vibrations of -OH groups. No significant wavelength shift of Em was observed in the Em-Cs composite film, and the spectrum of the Em-Cs composite film exhibited a peak pattern similar to that of Cs. These findings align with the results reported by Qin et al. (2020) [48], where no distinct peaks were observed in the composite film, indicating that no new chemical bonds formed between Cs and Em after Em addition to the chitosan solution. Consequently, the structure of the Em-Cs membrane remained unchanged following Em incorporation, indicating that the interaction between Cs and Em is of a physical nature. The slight variation in peak intensity is attributable to non-covalent interactions and hydrogen bonding between polymers within the film-forming solution.

#### 3.4.3. XRD Analysis Results

**Figure 5:**
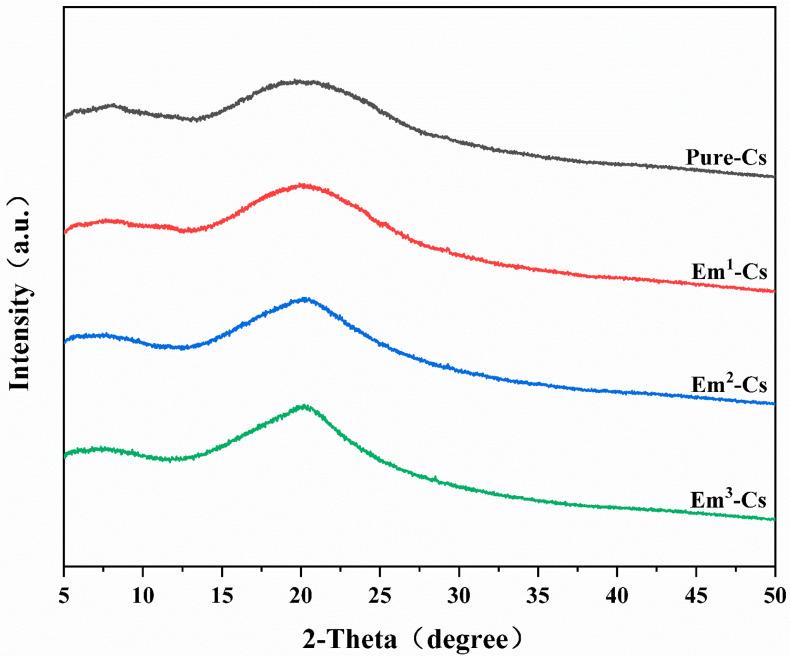
X-ray diffraction patterns of Chitosan and Emodin-Chitosan composite films.

According to previous research, the crystallinity index strongly influences the mechanical properties of the film [49,50]. Therefore, X-ray diffraction analysis was employed to determine the 2θ range from 5 to 50°, revealing the effect of Cs addition on the crystalline structure of the Cs film. The X-ray spectra of the Cs and Em-Cs composite films are shown in Figure 5.

Consistent with the amorphous nature of chitosan films, a broad characteristic peak was observed around 20° in all samples, reflecting a similar structural state as previously documented [51]. Upon adding Em, the peak intensified, demonstrating enhanced crystallinity. However, the characteristic peak of Em did not appear in the X-ray diffraction pattern, likely due to its low concentration, where Cs’ characteristic peak obscured Em’s peak. The increased crystallinity induced by Em addition accounts for the improved mechanical properties of the film, consistent with reports that low crystallinity leads to inferior mechanical performance in thin films, as also corroborated by related findings [24,50].

#### 3.4.4. Thermal Properties

**Figure 6:**
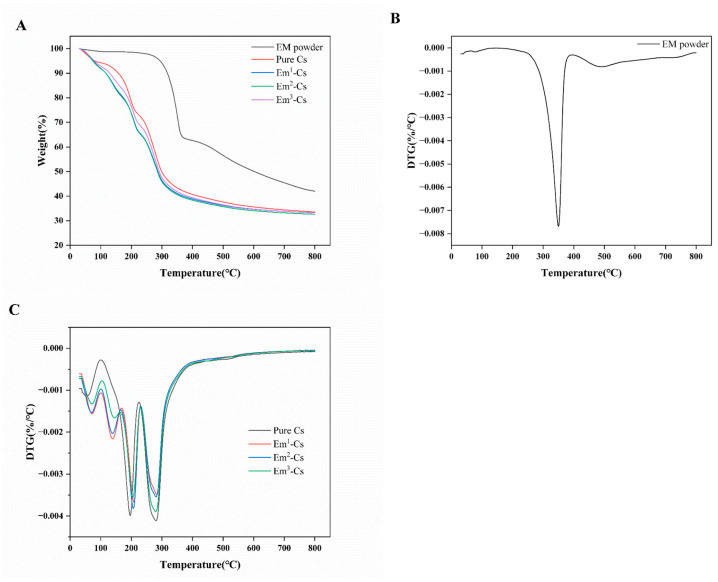
TGA thermal analysis curves for Emodin powder, pure Chitosan, and Emodin-Chitosan composite films (**A**); DTG thermal analysis curves for Emodin powder (**B**), pure Chitosan, and Emodin-Chitosan composite films (**C**).

Thermal gravimetric analysis (TGA) and derivative thermogravimetry (DTG) curves for Cs powder and film are shown in Figure 6A,C. Cs underwent two-step degradation, with the primary step occurring between 300 and 500 °C. The DTG curve confirms Em’s maximum weight loss temperature (Tmax) at 350 °C (Figure 6B). This weight loss is attributed to the fracture and decomposition of Em’s molecular backbone. The second step occurs around 500 °C, where the main chain of Em decomposes into smaller molecules of carbon, hydrocarbons, and carbon black. Ultimately, 42% solid residue is formed (Figure 6A). Unstable intermediates formed during the primary degradation process decompose rapidly [48].

All composite films underwent four-step degradation at temperatures of 30–100 °C, 100–170 °C, 170–230 °C, and 230–400 °C, respectively. The first weight loss step may have primarily resulted from the evaporation of free water within the film. The second and third steps may be attributable to the evaporation of bound water and glycerol. The predominant weight loss occurred between 230 and 400 °C, with maximum weight loss observed around 280 °C. This may be chiefly attributable to the degradation of Em and Cs polymer chains. The decomposition of emodin (Em) occurred at temperatures exceeding the preparation temperature of the Em-Cs composite film, which may suggest that Em is likely to be effectively embedded within the Cs film [52]. At 800 °C, the final residual mass of all films was approximately 30%. Among these, the Em3-Cs composite film exhibited the highest residual mass (33.2%). This demonstrates that the thermal stability of the film improves with the addition of Em.

### 3.5. Study on the In Vitro Antibacterial Properties of Em-Cs Composite Films

**Figure 7:**
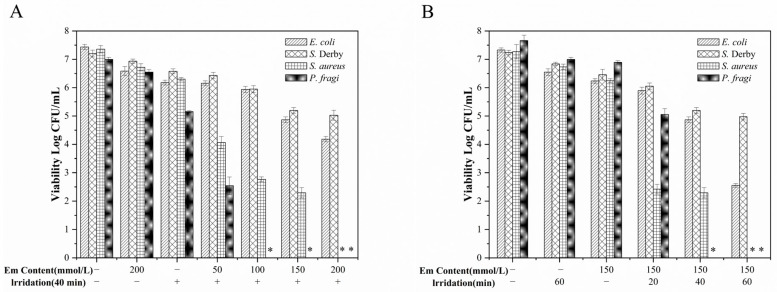
Antibacterial activity of the pure Chitosan and Emodin-Chitosan composite films against *Escherichia coli*, *Salmonella* Derby, *Staphylococcus aureus*, and *Pseudomonas fragi* in different Emodin content (**A**) and irradiation duration (**B**) conditions. * Indicates absence of growth.

After adding bacterial suspensions to the film, its antimicrobial efficacy was assessed by comparing the total colony counts of three foodborne pathogens (*E. coli*, *S.* Derby, and *S. aureus*) and one spoilage bacterium (*P. fragi*) under different treatment conditions. The specific experimental groups comprised: pure Cs film without blue LED irradiation (L − E−), pure Cs film with blue LED irradiation (L + E−), Em-Cs film without blue LED irradiation (L − E+), and Em-Cs film with blue LED irradiation (L + E+). The experimental results are presented in Figure 7.

As shown in Figure 7A, the presence or absence of blue LED irradiation had little effect on the antibacterial performance of the pure Cs membrane, with neither exhibiting significant antibacterial activity against any bacteria. Similarly, the Em-Cs membrane without blue LED irradiation showed no significant antibacterial effect even at an Em concentration of 200 μmol/L. However, after blue LED irradiation (40 min), when the Em concentration in the Em-CS composite membrane reached 100 μmol/L, *P. fragi* was undetectable on the membrane, while the viable counts of *E. coli*, *S.* Derby, and *S. aureus* decreased by approximately 1.5 Log_10_ CFU/mL, 1.25 Log_10_ CFU/mL, and 4.6 Log_10_ CFU/mL, respectively. Microbial counts decreased significantly with increasing Em concentration, culminating in the complete inhibition of both *S. aureus* and *P. fragi* at 200 μmol/L. As the Em content increases, the antibacterial effect intensifies, a finding that is consistent with those reported by Su et al. (2021) [44].

We further investigated the effect of illumination time on the antibacterial activity of the Em-Cs composite film, with results presented in Figure 7B. As illumination time increased, the blue LED delivered higher irradiation doses, leading to a corresponding improvement in antibacterial efficacy. When illumination time extended from 20 min to 40 min, viable bacterial counts exhibited a marked reduction. By 1 h of illumination, no viable *S. aureus* or *P. fragi* cells were detected. The results indicate that photodynamically induced bacterial inactivation in food packaging correlates with both photosensitizer concentration and light dose. Previous studies have also reported similar findings [53].

**Figure 8:**
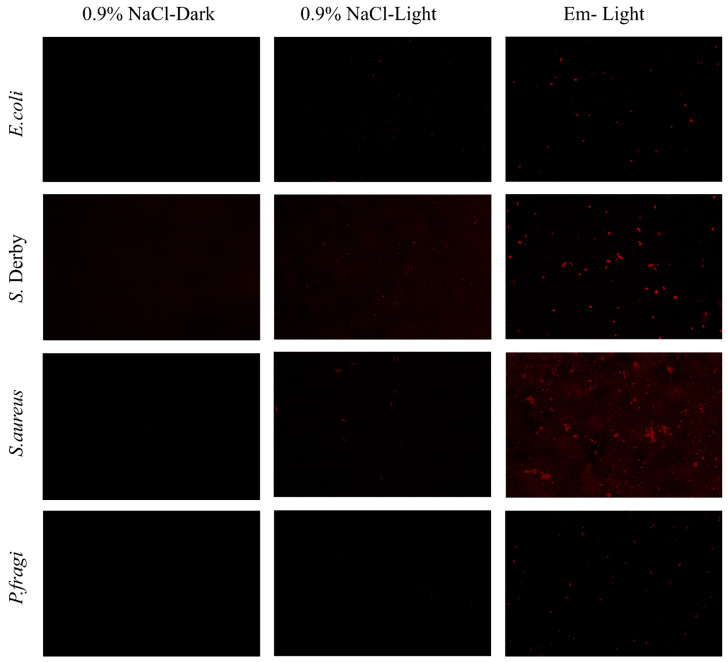
Fluorescence images of bacteria treated with the Emodin-Chitosan composite films, captured under a 450 nm wavelength light source or in darkness, following DCFH-DA staining.

In conjunction with the reactive oxygen species fluorescence imaging shown in Figure 8, under 450 nm blue light, Em molecules in the Cs matrix are excited to generate ROS, which disrupt bacterial cell membranes, DNA, and proteins. Consequently, the PDI-induced antibacterial film exhibits typical Em concentration and irradiation time-dependent characteristics [7]. Although Em may inhibit bacterial growth in darkness, under illumination conditions, the formation of photoproducts such as reactive oxygen species and singlet oxygen enhances antibacterial activity. Consequently, blue LED irradiation of the Em-Cs composite film generates reactive oxygen species, which may represent the bactericidal pathway of this composite film.

### 3.6. Analysis of Intracellular ROS in Bacteria

Photodynamic therapy primarily involves the excitation of visible light photons by photosensitizing agents exposed to specific wavelengths. Upon returning to their ground state, these photons transfer energy to oxygen molecules, thereby generating cytotoxic reactive oxygen species (ROS) [18]. This process inflicts irreversible damage upon bacterial cell membranes, DNA, and proteins [54,55]. Dichlorofluorescin diacetate (DCFH-DA) converts to green-fluorescent 2,7-dichlorofluorescein in the presence of ROS and is commonly employed to detect intracellular ROS generation in bacterial cells [56].

Figure 8 illustrates the effect of emodin on ROS production under visible light irradiation. Distinct green fluorescence was observed in *E. coli*, *S. aureus*, *S.* Derby *enterica*, and *P. fragi* treated with emodin and exposed to 450 nm light. In contrast, under dark conditions, the four bacterial strains treated with emodin exhibited behavior consistent with the control group (no fluorescence), indicating that emodin was unable to effectively induce reactive oxygen species (ROS) production in bacterial cells without 450 nm light excitation. Although literature reports that certain natural photosensitizers (such as curcumin and gallic acid) can induce ROS production in bacterial cells when used alone under non-light activation conditions [57,58], emodin did not exhibit this phenomenon.

### 3.7. Effect of Em-Cs Film Combined with 450 Nm Light Treatment on the Quality of Chilled Pork

To investigate the changes in viable bacteria during the storage of chilled fresh meat and the antibacterial performance of films on such meat, treated chilled fresh meat was placed at 4 °C under the following conditions: unpackaged, wrapped in pure commercially available cling film, wrapped in Cs film, and wrapped in Em-Cs composite film.

**Figure 9:**
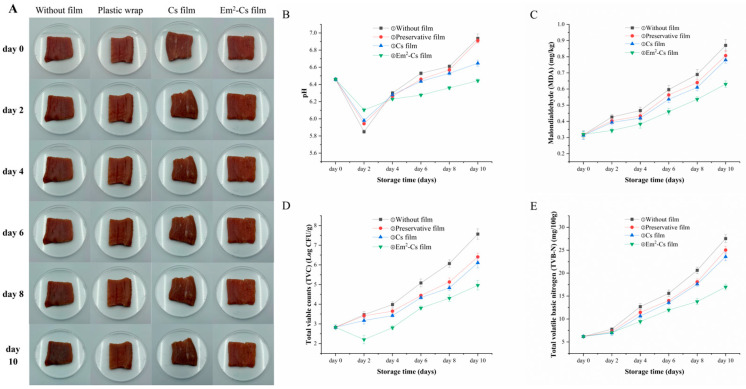
Images (**A**); pH (**B**); TBARS (Thiobarbituric acid reactive substances) values (**C**); TVC (Total viable counts) (**D**) and TVB-N (Total volatile basic nitrogen) (**E**) of chilled pork samples with different packaging groups during cold storage (4 °C).

The appearance, pH, TBARS value, TVC, and TVB-N of pork from different treatment groups are shown in Figure 9. The alterations in refrigerated pork samples from the four treatment groups over the 0–10 days are illustrated in Figure 9A. Compared to the control, the Em^2^-Cs coating significantly better maintained sample quality as storage time increased. The observed phenomenon is attributed to the synergistic bactericidal effect exerted by photodynamic therapy and the Em^2^-Cs composite membrane, wherein the Em^2^-Cs composite membrane serves as a carrier for the photosensitizer. Upon illumination, reactive oxygen species (ROS) such as ^1^O_2_ and O_2_^2−^ are generated [18]. This is confirmed by the TVC results in Figure 9D. Moreover, due to the oxidation of myoglobin, the surface of chilled fresh pork samples becomes dry and darkens in color after prolonged storage [59].

pH value serves as a key indicator of freshness in chilled pork, with its increase primarily attributable to microbial proliferation and protein degradation [60]. As microbial activity intensified during storage, the pH of chilled fresh pork (initially weakly acidic as seen in Figure 9B) increased over time, leading to ammonia production [61]. Compared to the control group, the Em-Cs group exhibited a relatively slower rate of pH increase. This may be attributed to the Em-Cs membrane combined with photodynamic treatment, exerting a killing or inhibitory effect on microorganisms [62]. Consequently, pork samples treated with the Em-Cs composite membrane demonstrated more stable pH characteristics, enabling sustained preservation of their chilled quality throughout storage.

The thiobarbituric acid reaction value serves as a key indicator for assessing lipid oxidation levels. As illustrated in Figure 9C, the TBARS value of fresh pork ranges between 0.29 and 0.32 mg MDA/kg. At a TBARS value of 0.5 mg MDA/kg, off-flavors become discernible in raw meat, whereas levels exceeding 0.6 mg/kg result in an unpleasant taste that is readily detectable by consumers [63]. During storage, TBARS values in all four pork treatment groups showed a progressive increase. The uncoated packaging group demonstrated persistently elevated values throughout the process, surpassing the 0.597 mg MDA/kg threshold as early as day 6, thereby exhibiting a substantial increase in comparison to the other groups. The TBARS value of the commercially available cling film group was found to be higher than that of the pure chitosan film group and the composite film group. Conversely, the TBARS value in the emodin-supplemented experimental group was lower than that observed in the pure chitosan film group devoid of emodin. The Em-Cs group persisted below the threshold until day 8, at which point it registered a mere 0.54 mg MDA/kg. By day 10, the TBARS value in the unpackaged group had escalated to 0.87 mg MDA/kg, whereas the Em^2^-Cs film group recorded a mere 0.63 mg MDA/kg. A comparative analysis suggests that lipid oxidation was effectively impeded in the Em^2^-Cs film group. Concurrently, TBARS values in both the Preservative film and Cs film groups remained consistently lower than those observed in the unpackaged control. These findings underscore the substantial efficacy of the Em-Cs composite film in suppressing lipid oxidation. Yang et al. (2024) reported that pork wrapped in chitosan-aloe-emodin films treated with light achieved malondialdehyde (MDA) levels of 5.787 mg/Kg, significantly lower than the MDA levels in control or chitosan-film-wrapped samples [12]. Furthermore, Li et al. (2024) reported that chitosan-gelatin films exhibited negligible DPPH radical scavenging activity, which increased with rising aloe emodin content [64]. These findings demonstrate synergistic antioxidant activity between the photosensitizer and chitosan.

To investigate the inhibitory effect of Em-Cs composite films on microbial growth in pork samples, the Em^2^-Cs composite film was selected as the experimental packaging material for chilled pork to suppress bacterial growth, with TVC measured. Figure 9D compares the total viable count under four packaging conditions, tracked over a 10-day storage period. The initial TVC of chilled pork was 2.83 log CFU/g. Over extended storage periods, TVC exhibited a progressive increase across all packaging conditions. A TVC value below 6 log CFU/g in fresh pork is considered acceptable to consumers [65]. Experimental results showed that unpackaged samples exceeded the TVC threshold of 6 log CFU/g by day 8, followed by those with commercial cling film and Cs film on day 10, while samples packaged with the Em-Cs composite film remained below the threshold at day 10. The results indicate that both Cs film and Em-Cs composite film effectively inhibit microbial proliferation in pork samples. However, the efficacy of Cs film is comparable to that of commercially available cling film, primarily attributed to the synergistic bactericidal effect of photodynamic treatment combined with Em-Cs film. This combination significantly reduced total colony counts during pork storage.

TVB-N serves as a key indicator for assessing meat quality and spoilage, reflecting its freshness [66]. According to the standard, when the TVB-N value in pork exceeds 15 mg/100 g, it indicates that the pork is no longer fresh [32]. As shown in Figure 9D, during storage, TVB-N values increased in all groups, with the unprotected control group exhibiting a significantly faster increase than the other groups. This is not only attributable to the synergistic bactericidal effect of photodynamic treatment and Em-Cs composite film, but may also be due to the barrier function of Em-Cs composite film against external environmental microbial growth factors such as water and oxygen [67]. On the sixth day, the TVB-N value of the unpackaged group reached 15.60 mg/100 g, exceeding the 15 mg/100 g threshold. The TVB-N values for the Cs membrane and Em^2^-Cs composite membrane groups on the eighth day were 13.80 mg/100 g and 13.76 mg/100 g, respectively, effectively extending the pork’s shelf life by 2 days. The TVB-N value for the Em^2^-Cs composite film treatment reached 16.97 mg/100 g by day 10. The addition of Em, coupled with light exposure, effectively inhibited microbial growth and reduced the accumulation of volatile basic nitrogen, thereby delaying the spoilage of chilled pork. This contributes to maintaining the quality and freshness of chilled pork over extended storage periods.

## 4. Conclusions

In this study, Em-Cs composite films were successfully fabricated via solution casting, with emodin as a natural photosensitizer uniformly dispersed in the chitosan matrix. Increasing Em concentration significantly enhanced film thickness, mechanical properties, and opacity, endowing the films with good applicability for food packaging. Under 450-nm light irradiation, the films generated ROS that effectively inactivated both Gram-negative (*E. coli*, *S.* Derby, *P. fragi*) and Gram-positive (*S. aureus*) bacteria; the photodynamic inactivation efficacy was dependent on Em concentration and irradiation duration. For chilled pork preservation at 4 °C, the film-light system reduced total viable count (TVC), delayed pH increase, inhibited lipid oxidation (TBARS), and lowered total volatile basic nitrogen (TVB-N) accumulation, extending the shelf life by 4 days compared with conventional packaging. This provides an eco-friendly, natural antimicrobial packaging strategy for perishable chilled meat.

Limitations of this work include the films’ insufficient water resistance (which may impair performance in high-humidity environments) and the relatively high cost of 450-nm light sources for large-scale cold chain applications. Future research will focus on optimizing film water resistance via surface modification, developing micro-light-integrated intelligent packaging, and expanding applications to other perishable foods.

## Figures and Tables

**Table 1:** Digital images and L*a*b* and ΔE values of the Chitosan film and Emodin-Chitosan composite films.

Sample Code	Pure Cs	Em^1^-Cs Film	Em^2^-Cs Film	Em^3^-Cs Film
Image	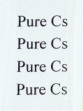	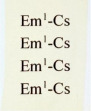	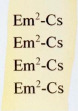	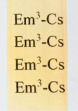
L*	94.43 ± 0.48 ^a^	94.13 ± 0.31 ^b^	93.540 ± 0.33 ^b^	92.54 ± 1.03 ^b^
a*	0.23 ± 0.04 ^a^	−1.6 ± 0.43 ^c^	−1.23 ± 0.31 ^c^	−0.82 ± 0.63 ^b^
b*	3.28 ± 1.11 ^d^	18.71 ± 4.94 ^c^	20.29 ± 1.15 ^c^	28.62 ± 2.32 ^a^
ΔE	4.36 ± 1.02 ^d^	19.13 ± 4.85 ^c^	20.55 ± 1.11 ^c^	28.67 ± 2.30 ^a^
Opacity	1.12 ± 0.03 ^d^	2.15 ± 0.05 ^b^	2.63 ± 0.04 ^a^	2.86 ± 0.07 ^a^

Data are presented as the Mean ± Standard Deviation. ^a,b,c,d^ Different superscript letters within the same row denote significant differences between samples (*p* < 0.05) (ANOVA). Pure Cs: Chitosan film without emodin; Em^1^-Cs film: Chitosan composite film with 100 μmol/L emodin solution; Em^2^-Cs film: Chitosan composite film with 150 μmol/L emodin solution; Em^3^-Cs film: Chitosan composite film with 200 μmol/L emodin solution.

**Table 2:** Physical and mechanical properties of Chitosan film and Emodin-Chitosan composite films.

Sample Code	Thickness (mm)	TS (MPa)	EAB (%)	MC (%)	WS (%)	WCA (°)	WVP (×10^−13^) (g/(m·s·Pa))
Pure CS	0.023 ± 0.02 ^b^	21.67 ± 4.29 ^c^	24.29 ± 0.51 ^c^	23.31 ± 2.74 ^a^	32.58 ± 0.35 ^e^	62.45 ± 0.78 ^d^	2.98 ± 0.26 ^a^
Em^1^-Cs	0.026 ± 0.01 ^a^	22.38 ± 0.78 ^c^	27.62 ± 0.42 ^b^	15.01 ± 1.07 ^b^	40.05 ± 0.40 ^a^	66.89 ± 0.48 ^c^	2.46 ± 0.32 ^b^
Em^2^-Cs	0.027 ± 0.03 ^a^	24.16 ± 2.74 ^b^	29.28 ± 0.86 ^a^	11.01 ± 0.56 ^c^	38.03 ± 1.53 ^b^	70.76 ± 0.64 ^b^	2.25 ± 0.25 ^c^
Em^3^-Cs	0.028 ± 0.04 ^a^	26.32 ± 1.25 ^a^	31.47 ± 0.47 ^a^	10.87 ± 0.72 ^c^	35.64 ± 0.31 ^c^	82.08 ± 0.57 ^a^	1.83 ± 0.26 ^d^

Date is presented as the Mean ± Standard Deviation. ^a,b,c,d,e^ Different superscript letters between columns indicate significant difference between the results (*p* < 0.05) (ANOVA). Pure Cs: Chitosan film without emodin; Em^1^-Cs film: Chitosan composite film with 100 μmol/L emodin solution; Em^2^-Cs film: Chitosan composite film with 150 μmol/L emodin solution; Em^3^-Cs film: Chitosan composite film with 200 μmol/L emodin solution.

## Data Availability

The original contributions presented in this study are included in the article. Further inquiries can be directed to the corresponding author.
